# Planarian Phototactic Assay Reveals Differential Behavioral Responses Based on Wavelength

**DOI:** 10.1371/journal.pone.0114708

**Published:** 2014-12-10

**Authors:** Taylor R. Paskin, John Jellies, Jessica Bacher, Wendy S. Beane

**Affiliations:** Department of Biological Sciences, Western Michigan University, Kalamazoo, MI, United States of America; United States Department of Agriculture, Beltsville Agricultural Research Center, United States of America

## Abstract

Planarians are free-living aquatic flatworms that possess a well-documented photophobic response to light. With a true central nervous system and simple cerebral eyes (ocelli), planarians are an emerging model for regenerative eye research. However, comparatively little is known about the physiology of their photoreception or how their behavior is affected by various wavelengths. Most phototactic studies have examined planarian behavior using white light. Here, we describe a novel planarian behavioral assay to test responses to small ranges of visible wavelengths (red, blue, green), as well as ultraviolet (UV) and infrared (IR) which have not previously been examined. Our data show that planarians display behavioral responses across a range of wavelengths. These responses occur in a hierarchy, with the shortest wavelengths (UV) causing the most intense photophobic responses while longer wavelengths produce no effect (red) or an apparent attraction (IR). In addition, our data reveals that planarian photophobia is comprised of both a general photophobic response (that drives planarians to escape the light source regardless of wavelength) and wavelength-specific responses that encompass specific behavioral reactions to individual wavelengths. Our results serve to improve the understanding of planarian phototaxis and suggest that behavioral studies performed with white light mask a complex behavioral interaction with the environment.

## Introduction

Planarians are non-parasitic flatworms that are an important model system for understanding stem cell biology [1]–[3], regeneration [4]–[6], toxicology [7], [8], and evolution [9], [10]. Additionally, with their true central nervous system and cerebral eyes connected to the brain, planarians have been used as a model for eye research. Several basic features found in planarian eyes are phylogenetically conserved such as photoreceptor cells containing opsin, a pigmented cup structure, and a host of eye-specific developmental genes that are essential for eye formation [11]–[14]. These common features, combined with the relative simplicity of the planarian visual system, make flatworms a valuable addition to the models used for investigating the basic features of eye biology and increasing our understanding of eye evolution and development.

**Figure 1:**
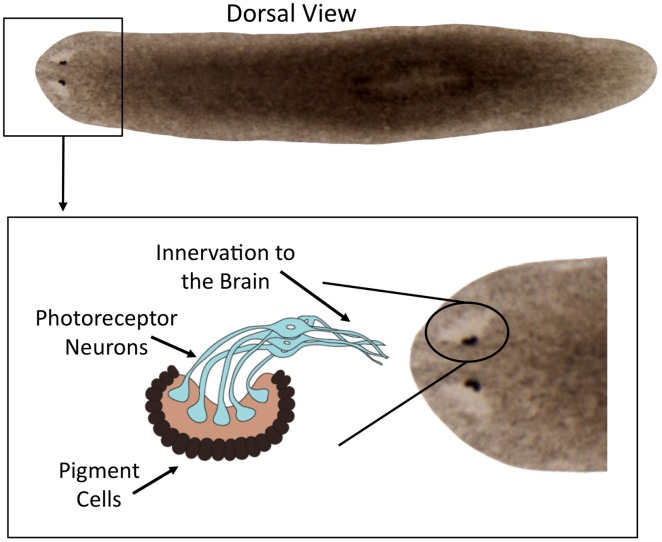
Planarian Eye Anatomy. The planarian species *Schmidtea mediterranea* was used. Boxed region shows a close up of the eyes, with an inset diagram of the light-sensing structures of the optic cup. The eye consists of two tissue types: the light capturing pigment cells and the photoreceptor neurons that transduce photons into signals sent to the brain.

Located on the dorsal side of the body, planarian eyes are composed of two cell types: pigment cells and photoreceptor neurons (Figure 1). The pigmented cells form a semi-lunar optic cup and function to absorb incoming light. Thus, each eyecup confers a left-right directional selectivity to visual information while the rostral location confers an anterior dimension to visual information transduced by the ocelli. The photoreceptor cells are bipolar neurons whose cell bodies are located outside of the optic cup [15]. Axons from the photoreceptor neurons project posteriorly into the brain, with some fibers forming a partial optic chiasma to integrate photosensory inputs from both sides of the animal [16], [17], [18]. The dendrites of the planarian photoreceptors extend inside the optic cup and form a rhabdomeric structure where opsin accumulates [12], [19]. Opsins are a highly conserved class of G-protein coupled receptors that covalently bond to a chromophore forming the visual pigment rhodopsin [20]. Transcriptome analyses reveal that the rhodopsin signaling pathway is conserved in planarians, including two R-opsin homologs [14].

Planarians are photophobic and when exposed to light they seek cover [21]–[23]. This negative phototaxis has been used to evaluate regeneration of the visual system [23]–[26], as well as memory storage and transference [27], [28]. In these planarian behavioral studies, analyses have been conducted with white light, which consists of an amalgamation of multiple wavelengths. However, many animals have been shown to have different behavioral responses to different wavelengths of light. For example, zebrafish larvae will swim toward ultraviolet (UV), blue, and red light but are only weakly attracted to green light [29]. Conversely, leeches detect and exhibit complex negative phototactic responses to UV and green wavelengths, with UV producing the maximal response [30], [31]. In *Drosophila* larvae, exposure to blue, violet, and UV wavelengths elicits negative phototaxis, while green and red light produces no behavioral response [32]. Similarly, the movement of *C. elegans* increases under blue or shorter wavelengths of light, again with maximum responses to UV [33].

A further complication of using white light for phototactic studies is that different sources of white light (e.g. halogen, Light-Emitting Diode or LED, and fluorescent) have varying spectral compositions. Even within a single source, such as the commonly used halogen light, substantial differences exist in the wavelengths included [34]. Additionally, regulation of intensity by controlling current also alters the spectral composition, giving rise to yet another poorly controlled variable. Therefore, we suggest that use of white light to study planarian photophobia may mask important behaviors associated with different wavelengths of light. We hypothesize that rather than a general photophobic response, planarians have differential responses across a range of wavelengths both within and outside of the visible spectrum. Here, we describe a novel planarian behavioral assay developed to test behavioral responses to individual wavelengths including UV and infrared (IR), which to the best of our knowledge have not previously been examined in these flatworms. Our data show that planarians display a complex, hierarchal photophobic response to specific ranges of wavelengths, in addition to a brief general response that appears to be more wavelength-independent. Furthermore, similar to leeches and *C. elegans*, planarians display the most robust responses to UV wavelengths. These results serve to improve our understanding of the basic biology of planarian eye function and suggest a previously underappreciated visual richness in these animals.

## Materials and Methods

### Colony Care

Asexual *Schmidtea mediterranea* were maintained as previously described [35], except worm water was comprised of 0.5 g/L of Instant Ocean salts. 6–9 mm worms were starved at least one week prior to experimentation before use.

### Light Sources

Ambient lighting was generated by directing two 100 watt LED flashlights onto the walls on either side of an otherwise completely dark room to produce diffuse background illumination of 50–55 lux (“Light Meter–Lux Measurement Tool” Version 1.2, iPhone application). LED wands (fixed resistor and RCA plug attached to a 9 volt battery with switch) were constructed as previously described [30], [31]. Each wand delivered roughly equivalent numbers of photons cm^−2^s^−1^ (flux), with the following nominal wavelength ranges: near IR (700–850 nm), red (615–640 nm), green (515–520 nm), blue (460–470 nm), and 2 wavelengths of near UV (395–405 nm and 360–365 nm). White light was obtained using a standard LED fiber optic illuminator with goosenecks from a dissecting scope setup. Approximate relative luminosity in the testing dish was assessed using a phototransistor coupled to a 2 mm diameter fiber optic [36]. As expected, intensity was greatest in quadrant 1 (Q1) and steadily decreased, with quadrant 4 (Q4) being the darkest. For the avoidance assay, commercially available red, green, and UV laser pointers with nominal peak wavelengths of 650 nm, 532 nm, and 405 nm (+/−10 for all) were used. In order to obtain a spot of light that was smaller than the worm itself, a piece of tape was placed on the end of the laser and punctured to create a pinhole that produced a circle of light approximately 2.5 mm in diameter.

### Photophobia Assay

A rectangular 7.6 cm×3.4 cm×1.1 cm testing dish, made from the top of a standard coverslip box, was placed over a sheet of white paper containing a template marked with the perimeter of the testing dish (for dish placement) and lines dividing the dish into four equal quadrants (1.9 cm×3.4 cm). There was also a half circle at the origin, with its apex midway through Q1, for directing light placement. LED wands were secured above the testing dish with a clamp attached to a ring stand, while a second clamp secured the battery pack to prevent unintended movement of the wand. The end of the LED wand was positioned about 5 cm above the top of the testing dish with the light directed into the half circle in Q1. An SLR camera was positioned over the testing dish using a tripod. On each experimental day, batteries were replaced in both flashlights and the LED wand. The testing dish was filled to a depth of 0.5 cm with worm water for each trial and emptied and wiped clean between wavelength presentations. In a single day, one wavelength was applied to total of 60 worms (10 groups of 6 worms, or 10 trials), repeated 3 times. For each trial, all worms were placed into Q1 before the camera was turned on. Except for controls, the light was switched on (time 0) at 5 seconds after recording started. Behavior was recorded for 2 minutes. Animals were allowed to rest at least overnight before the next wavelength. Wavelengths were generally tested in the order: control, IR, red, green, blue, UV 395, and UV 360.

### Neutral Density (ND) Filters

Filters used were 25.4 mm diameter nickel chromium coated fused silica (7980) as previously described [30], [31]. A holder was designed from stiff foam pipe insulation to position the LED wand above the filter such that all emitted light passed through the filter. ND filters attenuating 95% of light (optical density = 1.3) and 99% of light (optical density = 2.0) were used.

### Avoidance Assay

White paper was placed on the microscope stage so that laser light could be seen. A 100 mm Petri dish filled with 20 mL of worm water was positioned over the paper, and the microscope base’s brightfield light was turned to the lowest setting that allowed for recording. Individual worms were transferred to the middle of the dish and recording was started when the worm began traveling on the bottom of the dish. The laser beam was directed in front of the animal at a distance equal to one diameter of the circle of light (approximately 2.5 mm). Only a single wavelength was tested each day (with 30 worms repeated twice, for a total of 60 trials), and animals were allowed to rest at least overnight before the next wavelength (in the following order: red, green, and UV).

### Imaging and Recording

For the photophobia assay, imaging was done using a Canon EOS Rebel T5i SLR camera mounted to a tripod. For the avoidance assay, imaging was done using a Zeiss V20 fluorescent stereomicroscope with AxioCam MRc camera and Zen Lite software. Recordings from all behavioral trials were examined using Windows Media Player.

### Assay Analyses and Statistics

For the photophobia assay, the three repeat trials for each group were first averaged to compensate for individual animal variability. When determining location, at least 50 percent of the worm had to be in the quadrant. To examine the location of worms across all quadrants, all wavelengths were compared using a Kruskal-Wallis test, with Dunn’s Q corrected for tied ranks. The escape index was calculated as 1−(number of worms in Q1 at time X/number of worms in Q1 at time 0), and significance was determined using two-way repeated-measures ANOVA. A Bonferroni post hoc multiple comparisons test was conducted to examine differences between means. P≤0.01 was considered significant for all tests.

## Results

### A Novel Planarian Photophobia Assay to Test Responses to Individual Wavelengths

Planarian flatworms possess a well-documented negative phototactic (photophobic) behavioral response in the presence of light, as tested using various sources of multi-wavelength “white” light [22]–[26], [37], [38]. However, from available data, it is unclear whether planarians have a single general photophobic response or if their behavioral responses actually vary by wavelength as has been shown in other animals [29]–[33], [39]. To distinguish between these possibilities, we developed a novel behavioral assay (Materials and Methods). Because the LED wand was exchangeable, our setup allowed not only for testing behavioral responses to different visible wavelengths, but provided a means to investigate planarian responses to ultraviolet (UV) and infrared (IR) wavelengths as well.

**Figure 2:**
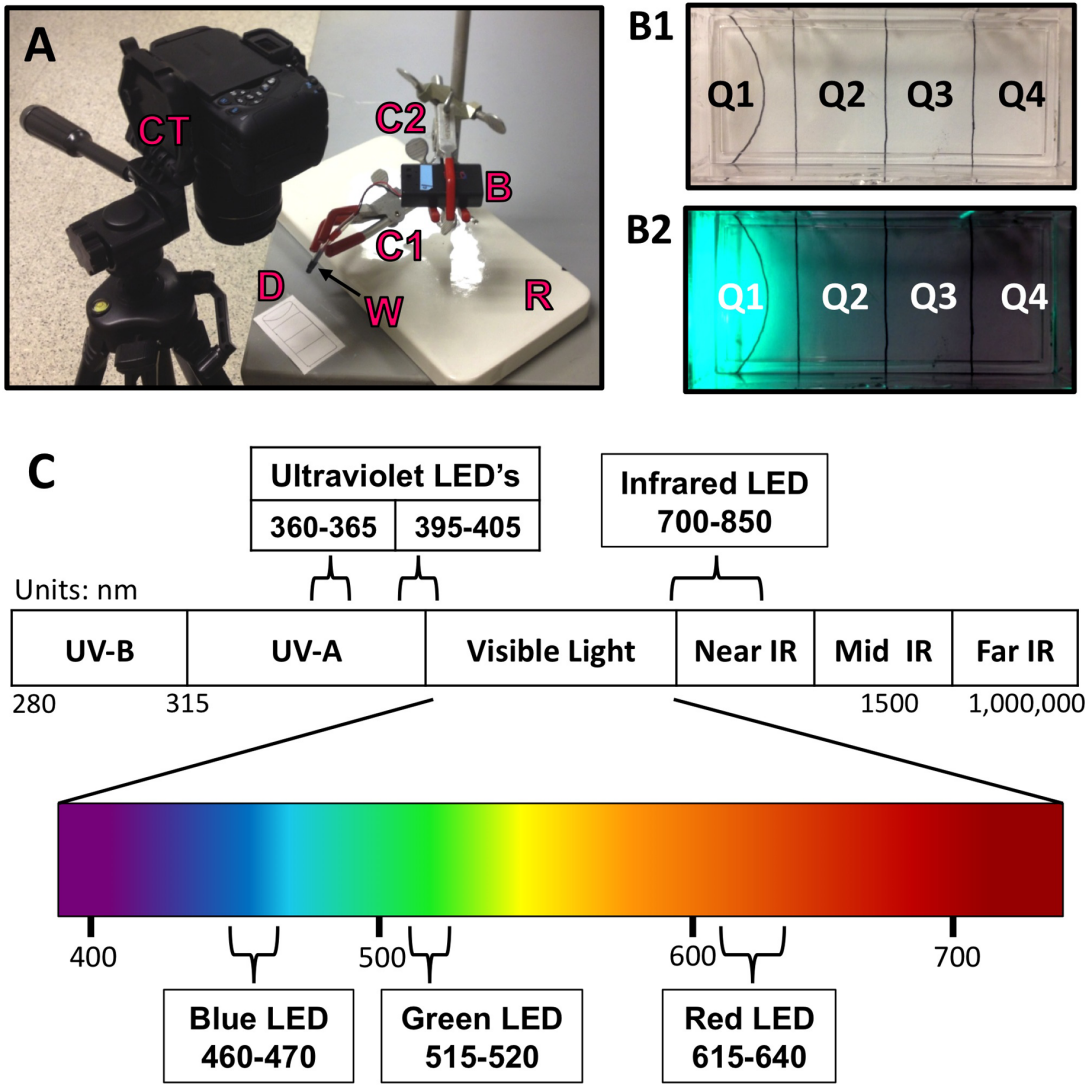
Photophobia Assay. (**A**) The imaging setup. CT = Camera mounted on tripod. W = LED wand. D = Testing dish. C1/C2 = Clamps. B = Battery pack. R = Ring stand. (**B**) Close-up of testing dish. (B1) The labeled guide placed underneath the dish marks the 4 quadrants (Q1–Q4) and the semi-circle where the LED light will be directed. (B2) Image of testing dish during a trial, showing the resulting light-to dark gradient. (**C**) The spectral composition of the LEDs used, and their location on the electromagnetic spectrum. UV = Ultraviolet. IR = Infrared.

One objective was to establish an easily reproducible photophobia assay with standardized testing parameters in order to improve comparability. Therefore, each LED wand was clamped above the testing dish at a fixed distance of about 5 cm (Figure 2A). Additionally, a sheet of white paper was placed beneath the testing dish, with four equal quadrants (Q1 to Q4) demarked (Figure 2B). To verify that the amount of light gradually decreased from Q1 to Q4, the intensity of light in each quadrant was estimated with a phototransistor. Finally, the assay used easily-constructed LED wands powered by 9 volt batteries, as previously described [30], [31], which allowed for some control of the ranges of wavelengths tested. Each wand was also designed to deliver roughly equivalent numbers of photons cm^−2^s^−1^ (flux) [30], [31]. For our experiments, the nominal wavelengths used were (Figure 2C): near IR (700–850 nm), red (615–640 nm), green (515–520 nm), blue (460–470 nm), and two distinct wavelengths of near UV light (395–405 nm and 360–365 nm). In addition, we also tested worm responses to white light using a standard LED fiber optic illuminator (with goosenecks) as typically used with a dissecting scope. The use of white light, even though there are certainly different spectra involved using LED or halogen sources, allowed us to compare responses from more restricted and narrow ranges of wavelengths with the non-specific white light typically used in planarian photophobia studies.

The photophobia assay was performed under ambient background lighting of approximately 50 lux (“no light” or controls), which was just sufficient to allow photography without agitating worms but not be completely dark. For the assay, the behavioral responses of 60 worms were tested (in 10 groups of 6 worms) for each wavelength (a single trial). Trials were repeated 3 times and the data averaged, to compensate for variability in individual worm responses. Trial parameters were as follows: camera recording was turned on, a group of 6 worms was placed in Q1, after 5 seconds the LED wand was turned on, and behavior was recorded for 2 minutes (the initial time was scored as when the light was first turned on). The 2 minute assay length was chosen based on preliminary data indicating the average time for worms to traverse the testing dish was ∼45 seconds (n = 36). Because of the remote possibility that the brief exposure to very weak UV light might cause damage, UV trials were performed last. Generally, worms were tested in order from longest to shortest wavelengths.

### Planarian Behavioral Responses Varied by Wavelength

**Figure 3:**
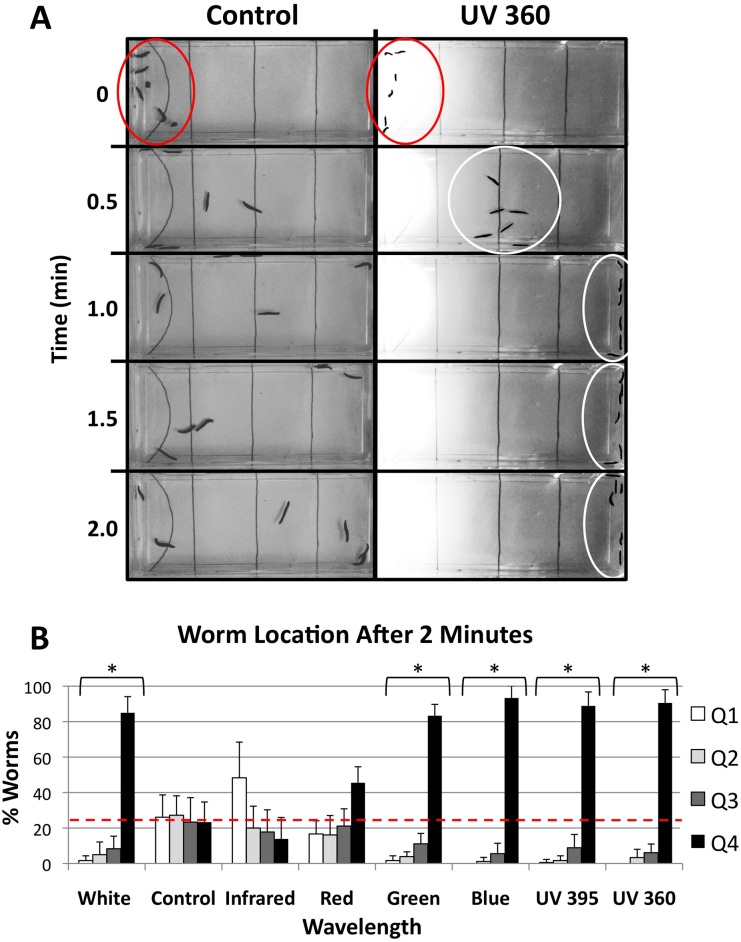
Planarian Photophobic Responses Vary by Wavelength. (**A**) Images of the photophobia assay showing single trials (one group, n = 6) for control (ambient light, left) and UV 360 (right) wavelengths. All worms begin in quadrant 1 (Q1, red circles). While control worms randomly explore the dish, in UV 360 trials worms move rapidly away from the light (white circles). Images enhanced for visualization. (**B**) Graph showing overall photophobic responses for each wavelength, as measured by worm location in each of the four quadrants (Q1–Q4) after 2 minutes. Photophobic responses are indicated by increased presence in Q4 (black bars) which is farthest from the light. Significance (asterisks = p<0.001 as compared to controls) was calculated by Mann-Whitney (with Dunn’s Q), which takes into account worm location across all four quadrants simultaneously. Red dashed line = average control value.

Using the above parameters, we performed our photophobia assay with control (ambient light only), IR, red, green, blue, and UV (395 nm and 360 nm) wavelengths, as well as with white light (Figure 3). Worm location by quadrant was scored at 30 second intervals (Figure 3A), with photophobia being assessed after 2 minutes (Figure 3B). Statistical significance (asterisks in Figure 3B) was assayed for the overall pattern of worm location throughout the entire dish (across all four quadrants), rather than for individual quadrants. Control groups explored the dish in an apparently random manner (Figure 3A and Video S1), such that by 1 minute animals were evenly distributed between all quadrants and remained so for the duration of the trial (with an average of 24.75% of worms in each quadrant at 2 minutes). This random exploration is consistent with initial exploratory behavior in new environments previously noted in planarians [40]–[42].

In contrast, exposure to green, blue, and both UV wavelengths resulted in strong photophobic responses, such that the majority of worms (≥80%) ended up located in the darkest quadrant (Q4, black bars in Figure 3B). In most of the UV trials, the worms congregated on the wall of the dish furthest from the light (Figure 3A and Video S2). As expected, worms exposed to white light also displayed strong negative phototaxis, with a striking correlation across all quadrants between white light (Q1: 1.67%, Q2: 5.00%, Q3: 8.33%, Q4: 85.00%) and green light (Q1: 1.67%, Q2: 3.89%, Q3: 11.11%, Q4: 83.33%). On the other hand, neither of the IR or red wavelength responses were statistically different from controls by the end of the trial (Figure 3B). Although the (small) majority of worms exposed to red wavelengths were in fact located in Q4 farthest from the light, worm location compared to controls was not statistically significant across all quadrants (p>0.20). Interestingly, although there was also no statistical significance in the location of worms exposed to IR across all quadrants as compared to controls (p≥0.50), a reverse trend was observed where the majority of worms were located in Q1 directly under the light (Figure 3B). Overall, these results suggest that our novel planarian photophobia assay is able to recapitulate the strong photophobia previously demonstrated by other methods.

**Figure 4:**
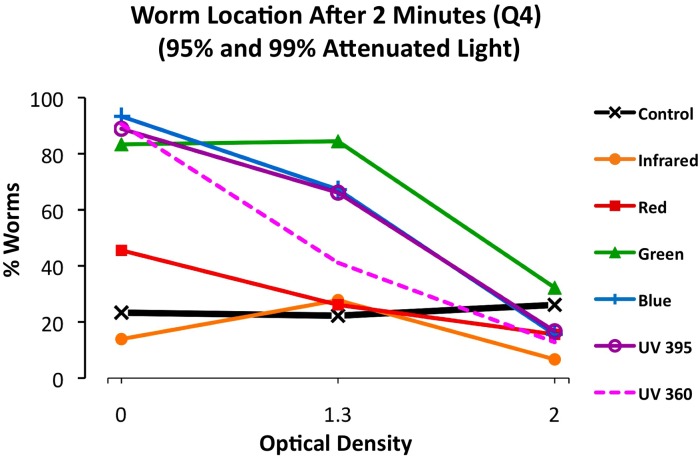
Photophobic Responses Result from Light Stimulus. Graph showing behavioral responses over increasingly attenuated light, as measured by the number of worms in Q4 at 2 minutes. Worms were exposed to full light, 95% attenuated light, and 99% attenuated light (or optical densities of 0, 1.3 and 2.0 respectively). The trend shows that phototactic responses decreased along with diminished behavioral stimuli (light).

To confirm that the observed behavioral responses resulted from visual detection of specific wavelengths and not other variables such as heat or nociception, we repeated our photophobic assay with neutral density filters. If responses to light are in fact a result of visual detection, we would expect worm responses to diminish in a predictable fashion as light attenuation increases (and the behaviorally relevant stimulus decreases). For the first trial, all LED lights were attenuated to 95%, so that only 5% of the light reached the testing dish, while in the second trial 99% of the light was attenuated (Figure 4). The results confirmed that the number of worms displaying photophobia steadily decreased with increased light attenuation, suggesting that the behavioral responses were the result of visual responses to specific ranges of wavelengths and not uncontrolled variables.

### Planarians Displayed the Severest Escape Responses to UV Light

**Figure 5:**
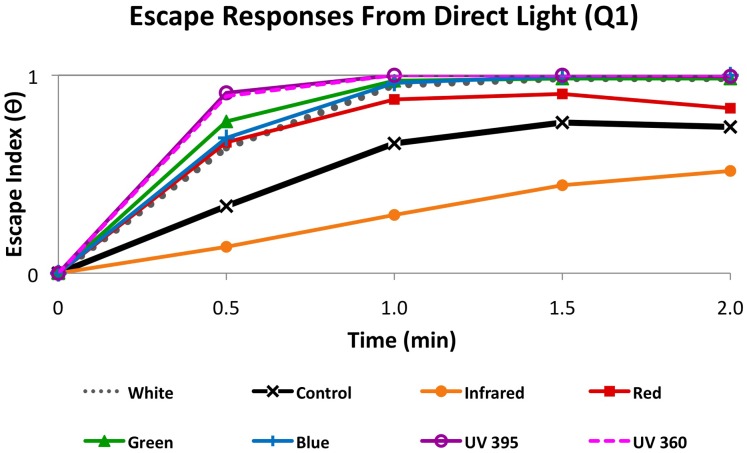
Escape Responses Vary by Wavelength. Graph showing escape responses as a measure of the severity of phototactic behavior. The escape index (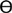) is based on the number of worms that leave Q1 (direct light), where a value of 1 indicates all worms have left Q1. Thus, higher values indicate stronger photophobic responses. At 30 seconds, the data indicate that UV wavelengths elicited a significantly stronger escape response, distinct from both controls (p<0.001) and all other wavelengths (p<0.01); while IR wavelengths produced an opposite, attractive response (p<0.001). All time points are significantly different from controls (p<0.001) by two-way ANOVA, except for red at 1.5 minutes (p<0.01) and 2 minutes (not significant). Note the latter data indicate by 2 minutes worms have returned to the direct red light source in Q1.

Although our data revealed that green, blue and UV light all resulted in robust photophobic responses (Figure 3B), we observed that worms exposed to near UV light appeared to move away from the light faster than for other wavelengths tested. This suggested that more complex differences exist between the photophobic responses than our scoring for photophobia at 2 minutes revealed. Thus, we next examined the rate at which worms escaped direct light (in Q1) by tracking both the number of worms that left Q1, and the number that returned, throughout the trial (Figure 5). To do this, we calculated an escape index (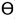), where 0 indicated all worms remained in Q1 while 1 indicated all worms had left Q1. Therefore, higher values represented stronger photophobic responses. It should be noted that an important difference exists between the analyses in Figure 3 and the analyses here in Figure 5 that represent how fast worms escape from direct light exposure. Because of this, the escape index as used here is a measure of the initial intensity of the response rather than a measure of overall strength of the response.

At 30 seconds, the escape indices for all wavelengths were statistically different (p<0.001) from controls (Figure 5), including red and IR (which were not significantly different in overall photophobic response (Figure 3). However, analyses revealed that escape responses to the UV light were significantly faster (p<0.01) than for all other wavelengths, confirming our observations that UV light caused the most extreme initial photophobic response. Additionally, the escape indices highlighted that reactions to green, blue, and white light at 30 seconds represented an intermediate behavioral response, which (while still strongly photophobic) was statistically different from both the UV responses (p<0.01) and the random exploration of controls (p<0.001). Interestingly, white light was more similar to (though not statistically different from) blue escape responses (Figure 5), in contrast to overall photophobic response (Figure 3) where white light was more similar to green. This may be related to the spectral composition of white light LEDs that typically contain several broad peaks, including notable amounts of energy in the blue range.

For IR light, the escape index (Figure 5) at all time points was significantly different from controls as well as all other wavelengths. This is in contrast to the overall photophobic response to IR light (Figure 3), which was not statistically different from controls even at the earlier 30 second time point. In particular, the escape index showed that IR wavelengths produced an opposite phototactic response, where worms were initially more likely to remain under direct light (Q1) than controls. This suggests the possibility that planarian responses to IR might be slightly photopositive, a hypothesis that would first need to be investigated in much greater detail. These data also indicate that the planarian visual system may be able to respond to IR wavelengths in some as yet unknown manner.

Most surprisingly, at 30 seconds the escape index for red light was significantly different from controls (p<0.001), illustrating an early visual behavioral response that was not different from the intermediate response noted above for green, blue and white. This was particularly unexpected given that the overall photophobic response to red at 2 minutes was not different than controls (Figure 5). Closer examination of the escape responses to red light revealed that responses remained significantly different at 1 minute (p<0.001), and at 1.5 minutes (p<0.01), but were no longer statistically different from controls by 2 minutes (Figure 5). This reflects the observation that at 2 minutes, worms that previously left Q1 returned, despite the continued presence of the red light exposure. When overall photophobic response (Figure 5) across all quadrants was examined at earlier time points, this pattern of an initial photophobic response to red light that decreased over time was again observed: significant at 30 seconds (p<0.02) and at 1 minute (p<0.01), marginal at 1.5 minutes (p<0.05), and not significant at 2 minutes. These data suggest that after an initial photophobic response worms subsequently stopped responding to red wavelengths.

### Planarians Have Both General and Wavelength-Specific Photophobic Responses

**Figure 6:**
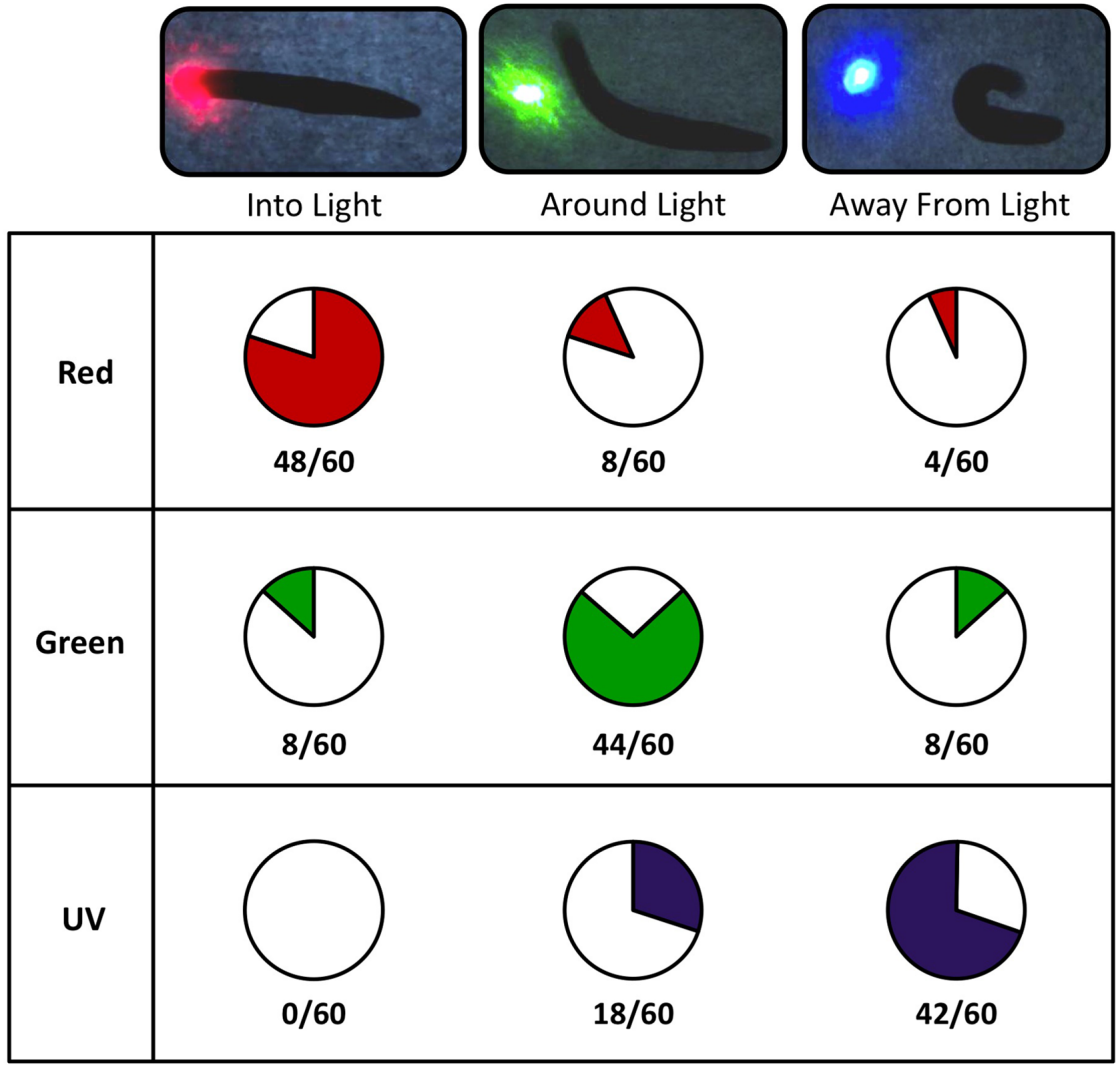
Light Avoidance Responses Vary by Wavelength. Avoidance assay to test worm responses when approaching areas of direct light. Red (top row), green (middle row), and UV (bottom row) wavelengths of laser light were placed in the worm’s path (photos), resulting in three distinct behaviors (shaded areas): worms moved into the light (left column), went around the light (middle column), or avoided the light by making 90–180 degree turns (right column).

The overall photophobic response data, combined with escape index analyses, suggested that while planarians displayed different responses to different wavelengths (with UV causing the most robust responses), there may also exist a separate, wavelength-independent photophobic response to being placed under direct light such as might be expected with broadly-tuned visual pigments. In order to test this idea, we examined avoidance responses to different wavelengths (Figure 6). Whereas previously we examined whether or not planarians would move away from light exposure, our avoidance assay tested the reverse behavior: whether or not worms would choose to enter a light source. However, the LED wands we used in our previous assay produced a field of light that was too large to record worm movement from outside the field into the light. Therefore, we switched to the use of tiny spots of laser light under high magnification (under a stereomicroscope). We covered the end of a laser pointer with a piece of tape that had a single pinhole in the center, thus obtaining a much smaller coherent circle of light. For illustration, compare the relative size of the light field versus a single worm in our photophobia assay (UV 360 panels in Figure 3A) and in our avoidance assay (Figure 6). We chose red, green and UV laser lights as representative of the range used in our photophobia assay. We expected that if wavelength-specific responses existed, worms would respond with increasing severity to avoid entering regions lit by red, green and UV wavelengths, respectively.

To test for light avoidance, the laser was pointed directly in front of a worm’s path at a distance roughly equal to one diameter of the circle of light. This ensured that worms began the assay outside the direct light source but was close enough that worms continued moving in the direction of the light. Three distinct behaviors were observed. As worms approached the light source they either 1) did not respond and continued moving directly into the light, 2) moved around the light by making a slight directional change to one side without crossing into the light, or 3) abruptly made a 90–180 degree turn in the opposite direction of the light (photos in Figure 6). Consistent with our previous data, when exposed to the red laser the majority of worms (80%) were not affected and continued moving directly through the light (top row of Figure 6, and Video S3). When confronted with the green light, the majority (73.33%) of worms chose to go around either the right or left side of the light without entering the most luminous spot (middle row of Figure 6, and Video S4). Strikingly, as worms approached the UV light their reaction was even more dramatic with 70% of the animals suddenly changing direction at a 90–180 degree angle in order to avoid the light and directing movement away from it (bottom row of Figure 6, and Video S5). Furthermore, not a single worm chose to travel into the UV light, even though 13.33% of worms did so with green light.

**Figure 7:**
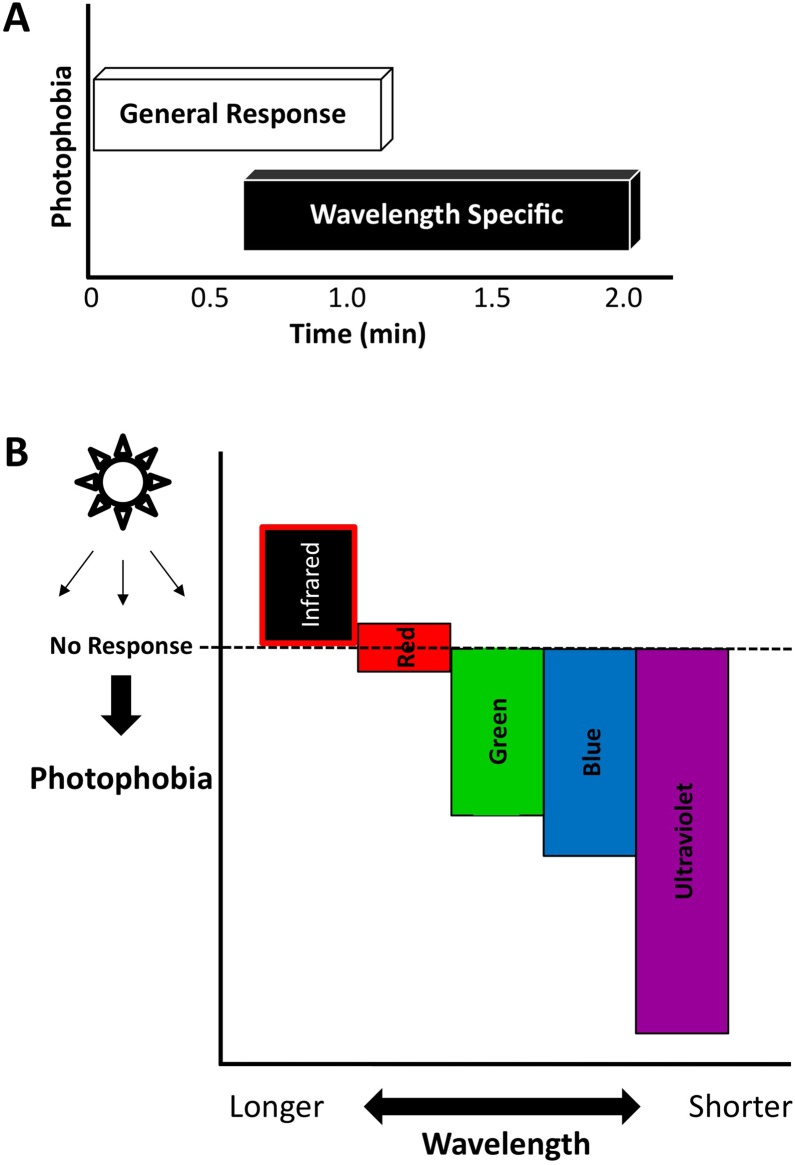
Planarian Photophobic Behavior is Hierarchal. (**A**) Graph showing the likely relationship between the two types of photophobic responses uncovered by our data: the general photophobic response, which occurs immediately after exposure to any wavelength, and the wavelength-specific responses. (**B**) Graph depicting the inverse relationship between photophobic responses and wavelength.

These results are consistent with our previous data showing that planarians exhibited differential responses to different ranges of wavelengths of light. They also confirmed that not only did UV light produce the strongest photophobic responses and most robust initial responses, but that an intermediate and less severe photophobic response occurs with wavelengths within the visible spectrum such as green. Furthermore, these results demonstrated that planarians lack a red wavelength-specific behavioral response, suggesting that the escape response we observed to red light reflects instead an initial wavelength-independent photophobic response (Figure 7A).

## Discussion

Our results support the hypothesis that planarians do possess differential behavioral responses to light across a range of wavelengths. Our data also reveal that planarian phototactic responses occurred in a behavioral hierarchy (Figure 7B), where the shortest wavelengths (in this case near UV light) caused the most intense photophobic responses while longer wavelengths produced no effect (for red) or even opposite effects (in the case of IR). Thus, an inverse relationship appears to exist between the wavelength and the intensity of the worm’s photophobia. These results highlight the importance of the spectral composition of light for planarian behavior and suggest that the current standard use of poorly characterized white light in planarian phototactic studies may mask more complex behaviors.

Unexpectedly, our data also suggested that planarian photophobic behavior may involve two different response types: a general photophobic response to luminal contrast (for example a rapid phasic change in luminosity) and more wavelength-specific photophobic responses (Figure 7A). The general photophobic response occurred immediately after light exposure and drove planarians to escape the light source regardless of wavelength (except for IR). This initial response may be due to the change in contrast that occurs when worms are suddenly exposed to light after leaving their preferred low/no light environment and presumes either broadly-tuned photopigments or some unknown aspect of phototransduction. In contrast, the wavelength-specific responses encompass specific behavioral reactions that vary depending on the wavelengths involved. The difference between the general and wavelength-specific responses can be seen in the planarian response to red light. Although worms displayed an initial general response to escape the light source, they quickly adapted to it in order to return into the direct light (Figure 5). This lack of a red-specific negative light response was confirmed in both our main photophobic assay (Figure 3B) and our avoidance assay (Figure 6). Together, these data illustrate that planarian photophobic behavior is complex and coordinated and not just the result of simple general light avoidance.

In this hierarchy, planarian responses to near IR light were the most surprising as worms appeared to be attracted to it. While worm localization across all quadrants was not statistically different from controls (illustrating a lack of photophobic response, Figure 3B), the escape indices for IR were significantly different at all time points (p<0.001, Figure 5) highlighting a slight but apparently real worm preference for remaining under direct IR light. The visual detection of IR has not been examined in planarians, although a few studies have shown that IR radiation causes increased stem cell proliferation [43], [44]. Our data seem to suggest that planarians may be able to detect IR by some mechanism. Although alternative explanations cannot be ruled out (for instance, IR may create a shadow effect by reducing the activation of opsin, thus making the IR quadrant appear darker than the ambient room lighting), IR detection is found in various parts of the animal kingdom. For example, some snakes and bats possess IR receptors called pit organs that are capable of sensing thermal stimuli [45]. Additionally, the visual systems of freshwater fish (such as the common carp, tilapia, zebrafish, green swordtail, and guppies) are also able to detect IR, an ability that may be directly related to their environmental conditions and/or circadian cycles [46–48]. The spectral absorption of water depends largely on the concentration of suspended particles such as dissolved oxygen and organic material, which enhance scattering and absorption of short- and mid-wavelengths [48]–[50]. Therefore, fish living in turbid water have sensitivity to slightly longer wavelengths [51]. IR detection could also be an adaption for nocturnally active animals as both moonlight and starlight consist of longer wavelengths [46], at least in very shallow water environments where there might be some IR penetrance.

Our data demonstrate that, like leeches and *C. elegans*, planarians are strongly photophobic to short wavelengths of light, with UV causing the greatest responses [30], [31], [33]. Differential responses to specific wavelengths are well documented in the literature and reveal that an animal’s sensitivity to each wavelength depends largely on its natural habitat and physiological needs. Fish that live in the ocean are typically most sensitive to blue wavelengths due to the fact that 470 nm blue light penetrates the greatest [48], [51]. For example, zebrafish are more positively phototactic to UV and blue than green light [29]. In contrast, the majority of flying or foraging insects are attracted to UV and green light, which they use in characterizing and identifying food sources [39], [52], [53]. For planarians, predator avoidance cues are likely to be the most crucial for survival, as they have few natural defenses and consist solely of soft tissues with no exoskeleton, venom, teeth, or claws. Thus, it makes sense that they would display strong photophobic behavior, particularly to daylight-related UV wavelengths. Furthermore, UV exposure causes significant damage to nucleic acids and proteins [54]; in planarians prolonged exposure to UV radiation damages their protective mucosal layer and leads to visible wounds [55]. Thus, a robust UV avoidance might offer a significant adaptive advantage.

UV detection is very common among animals, but the mechanisms used vary greatly. For example, several species of birds, fish, and insects have UV-sensitive photopigments [56], [57], while other animals use oil droplets or screening pigments [56], [58]. Additionally, it has been shown that when exposed to UV, invertebrate opsins can be converted to an intermediate that can regenerate the original UV opsin, which prevents bleaching and allows for continued detection of UV light [59], [60]. The damaging effects of UV exposure are so important that some animals also have general dermal methods to detect UV. *Drosophila* larvae possess neurons that cover their body wall and detect UV light using a chemosensory G-protein coupled receptor pathway [32] distinct from the more commonly understood photopigments. *C. elegans* detect UV using a receptor called LITE-1, which is a member of the invertebrate Gustatory receptor family [33]. The photophobic response to UV is so robust in *C. elegans* that illumination of only a few neurons causes behavior [33]. Extraocular detection of UV has also recently been discovered in the leech [30], [31]. Extraocular or dermal photoreception has been noted previously in planarians in the historical literature [61]. Confirming these reports, our initial behavioral observations found 98% of planarians (n = 15) tried to move away from UV light placed on the tail alone. Future experiments should focus on investigating the mechanisms involved, as these are currently unknown. In summary, our results strongly support the notion that visual responses in planaria may be more complex than previously understood.

## Supporting Information

Video S1 **Photophobia Assay Trial with Control/Ambient Light.**
(MOV)

Video S2 **Photophobia Assay Trial with UV 360 LED Light.**
(MOV)

Video S3 **Avoidance Assay Trial with Red Laser Light.**
(MOV)

Video S4 **Avoidance Assay Trial with Green Laser Light.**
(MOV)

Video S5 **Avoidance Assay Trial with UV Laser Light.**
(MOV)
